# Acute acalculous cholecystitis causing gall bladder perforation in children

**DOI:** 10.4103/0971-9261.72439

**Published:** 2010

**Authors:** Parag J. Karkera, Gursev Sandlas, Ritesh Ranjan, Abhaya Gupta, Paras Kothari

**Affiliations:** Department of Pediatric Surgery, Lokmanya Tilak Municipal General Hospital, Sion, Mumbai, India

**Keywords:** Acute cholecystitis, biliary peritonitis, gall bladder perforation

## Abstract

We report two cases of children who presented with acute abdomen due to gall bladder perforation and biliary peritonitis. Cholecystectomy with peritoneal lavage proved curative.

## INTRODUCTION

Gall bladder perforations (GBP) after cholecystitis are usually seen in elderly patients (>60 years) and are rare in children,[1] So far, <15 cases were reported in the literature, and most of them were associated with typhoid fever. We report two cases of acute abdomen in the pediatric age group, clinically diagnosed as perforative peritonitis and detected as GBPs at laparotomy.

## CASE REPORTS

### Case 1

An 11-year-old male child without any known medical comorbidity presented with a 3-day history of sudden-onset severe abdominal pain and distension. It was initially localized to the right upper quadrant and right lumbar region, but had evolved to a more generalized distribution at the time of presentation. It was associated with moderate-grade fever and nonbilious vomiting for 3 days.

His vital signs were stable, except for tachycardia. His abdomen was distended, with generalized tenderness, guarding on palpation and minimal movement with respiration. His bowel sounds were sluggish. The laboratory tests revealed leukocytosis and neutrophilia. His serum amylase, lipase and serum electrolytes were all within normal limits. Abdominal radiographs showed no signs of intestinal obstruction or pneumoperitoneum. Ultrasound of the abdomen showed mild ascites and rest of the small and large bowel loops, visualized pancreatic parenchyma, liver, biliary tree were reported as normal. With a clinical diagnosis of perforative peritonitis, probably enteric ileal or an appendicular perforation, an emergency laparotomy was performed.

At laparotomy, about 1/2 L of bilious fluid was drained. The gall bladder was found densely adherent to the duodenum. On adhesiolysis, a necrotic patch on the body of the gall bladder with a perforation was found. The anatomy of the Calot’s triangle was obscure and hence a near-total cholecystectomy, including the perforated area, was performed. Histopathology of the specimen showed a gall bladder lined by mucosa with focal ulceration and hemorrhage and the wall showing necrosis. A pathological diagnosis of acute on chronic gangrenous cholecystitis with perforation was made. Postoperatively, the Widal test was negative and the blood culture had shown no growth. The patient had an uneventful recovery.

### Case 2

An 11-year-old male presented with generalized abdominal pain for 1 day that had initiated in the right lumbar and suprapubic region. He also complained of fever and multiple episodes of nonbilious vomiting. There was no other significant past or present history. There was generalized tenderness and guarding on abdominal examination.

All blood investigations (hemogram, electrolytes, serum lipase, amylase, liver function tests) and radiograph of the abdomen were normal. The ultrasound abdomen showed free fluid in the abdomen with internal echoes. The bowel loops were dilated with sluggish peristalsis and pancreas, liver and biliary tree were reported as normal. With a clinical diagnosis of perforative peritonitis (appendicular or Meckel’s diverticular perforation) in mind, an emergency laparotomy was performed. Intraoperatively, frank bile was noted in the peritoneal cavity. The omentum was loosely adherent to the gall bladder, which had a perforation in the body. A fundus first cholecystectomy and peritoneal lavage was performed. Postoperatively, the Widal test was negative and the blood culture showed no growth. Histopathology revealed acute on chronic cholecystitis. The patient was discharged on the 7^th^ postop day and is doing well on follow-up.

## DISCUSSION

Only 5–10% of the patients with acute cholecystitis are associated with acalculous cholecystitis.[2] GBP occurs in 2–11% of acute cholecystitis patients.[1–4] It is more likely to be found in patients with recent severe trauma, critical illness, cardiovascular surgery or severe burns. The mortality rate is in the range of 12–16%.[5,6] GBP is a well known, although unusual complication, in enteric fever.[7]

Perforation results from occlusion of the cystic duct (most often by a calculus), which causes a rise of the intraluminal pressure due to retained intraluminal secretion. Our patients probably developed spontaneous GBP due to ischaemia of the gall bladder wall with inflammation and acalculous cholecystitis. Infections, malignancy, trauma and drugs (e.g., corticosteroids) and systemic diseases such as diabetes mellitus and atherosclerotic heart disease are common predisposing factors.[3] Fundus, followed by the body, are the most distal part with regards to blood supply and therefore this makes them the most common sites for perforation.[2,6]

Acute uncomplicated cholecystitis is more common among females, with a female to male ratio of 2:1. However, GBP is more frequent in the male gender.[4] GBPs are rare entities in children, with <15 cases being reported in the literature so far, and most of them were associated with typhoid fever.[7,8] Goel *et al*. reported a case in a 14-year-old, preoperatively correctly diagnosed as acalculous cholecystitis with GBP, which was confirmed on laparotomy.[9] Both our patients had no tests positive for enteric fever.

Perforation of the gall bladder can occur as early as 2 days after the onset of acute cholecystitis, or after a few weeks.[2] The perforation in both our patients had occurred within 72 h of the onset of symptoms.

Abdominal X-rays may not always show pneumoperitoneum, as seen in our patients, and hence they are not always helpful. Ultrasonography and computerized tomography (CT) may demonstrate abdominal fluid but lack specificity to diagnose GBP.[7] Significant ultrasound findings of gall bladder thickening (>3.5mm), distension, pericholecystic fluid and positive sonographic Murphy sign seen in cases of acute acalculous cholecystitis[10] may also be sometimes present in GBP, although none of them is very specific. The “HOLE” sign, in which the defect in the gall bladder is visualized, is the only reliable sign of GBP.[4] Sensitivity of CT in the detection of gallbladder perforation and biliary calculi has been reported to be 88% and 89%, respectively. These figures are higher than those reported for the ultrasonographic examination.[2] Other modalities used to detect GBP include diagnostic peritoneal lavage and retrograde cholangiography, HIDA scan.[9]

Early surgical intervention is an important step in the management of GBP. Although, clinically, it is difficult to predict the diagnosis of GBP, it is commonly assumed as bowel perforation when a patient presents with features suggestive of perforative peritonitis.[2,4,6] Cholecystectomy and drainage of an abscess, if present with peritoneal lavage, are usually sufficient as treatment. Laparoscopic cholecystectomy can be performed for acute, gangrenous and/or perforated cholecystitis as well as uncomplicated cholecystitis.[4]

Earlier reported Indian series of cholecystitis in children did not have any GBPs.[11] Our cases are unusual because our patients were children, with no prior history suggestive of gall bladder disease, and had no known medical comorbidity and showed absence of gall stones on surgery. Histopathological examination of the specimen showed features of acute on chronic cholecystitis leading to the derivation that the prior episodes of cholecystitis in these patients were clinically silent.

Early diagnosis of GBP and immediate surgical intervention are of prime importance in decreasing the morbidity and mortality associated with this condition. Similarly, given the clinical scenario, we believe that interval cholecystectomy in all diagnosed cases of acalculous cholecystitis should be a viable option to consider preventing future complications.
